# Photo- and X-ray Induced Cytotoxicity of CeF_3_-YF_3_-TbF_3_ Nanoparticle-Polyvinylpyrrolidone—“Radachlorin” Composites for Combined Photodynamic Therapy

**DOI:** 10.3390/ma17020316

**Published:** 2024-01-08

**Authors:** Alina I. Khusainova, Alexey S. Nizamutdinov, Nail I. Shamsutdinov, Svetlana Kalinichenko, Damir I. Safin, Marat Gafurov, Elena V. Lukinova, Sergey Kh. Batygov, Sergey V. Kuznetsov, Sergey V. Zinchenko, Pavel V. Zelenikhin, Maksim Pudovkin

**Affiliations:** 1 Institute of Physics, Kazan Federal University, 18 Kremlyovskaya Str., 420008 Kazan, Russiamichaeldermoon@gmail.com (N.I.S.); delfinchik1290@gmail.com (S.K.); safidami@mail.ru (D.I.S.); zinchenkos.v@mail.ru (S.V.Z.); pasha_mic@mail.ru (P.V.Z.); jaz7778@list.ru (M.P.); 2 Department of General Chemistry, Belgorod State National Research University, 85 Pobedy Str., 308015 Belgorod, Russia; elena.v.lukinova@gmail.com; 3 Prokhorov General Physics Institute of the Russian Academy of Sciences, 119991 Moscow, Russia; sbatygov@mail.ru (S.K.B.);

**Keywords:** photodynamic therapy, combined photodynamic therapy, Radachlorin, nanoparticle-photosensitizer conjugates

## Abstract

The Ce_0.5_Y_0.35_Tb_0.15_F_3_ nanoparticles with a CeF_3_ hexagonal structure were synthesized using the co-precipitation technique. The average nanoparticle diameter was 14 ± 1 nm. The luminescence decay curves of the Ce_0.5_Y_0.35_Tb_0.15_F_3_ nanoparticles (λ_em_ = 541 nm, ^5^D_4_–^7^F_5_ transition of Tb^3+^) conjugated with Radachlorin using polyvinylpyrrolidone coating as well as without Radachlorin were detected. Efficient nonradiative energy transfer from Tb^3+^ to the Radachlorin was demonstrated. The maximum energy transfer coefficients for the nanoparticles conjugated with Radachlorin via polyvinylpyrrolidone and without the coating were 82% and 55%, respectively. The average distance between the nanoparticle surface and Radachlorin was *R*_0_ = 4.5 nm. The best results for X-ray-induced cytotoxicity were observed for the NP-PVP-Rch sample at the lowest Rch concentration. In particular, after X-ray irradiation, the survival of A549 human lung carcinoma cells decreased by ~12%.

## 1. Introduction

For many years, mankind has been developing oncological disease therapies. One of the promising methods is photodynamic therapy (PDT) [1,2], which is based on the activation of photosensitizer molecules via visible light [3,4,5,6]. In particular, photosensitizer molecules can accumulate in the tumor, and being exposed to laser radiation, the photosensitizer molecules receive energy and transfer it to the oxygen and water molecules, turning it into excited states (so-called reactive oxygen species). Singlet oxygen can be classified as reactive oxygen species that are highly aggressive oxidizers [7,8]. As a consequence, these forms of oxygen can damage the vascular system of the tumor through cell necrosis or apoptosis [3,4]. However, visible radiation is not able to penetrate deeper into the tissues than several centimeters [9]. Moreover, its power density sharply attenuates due to several physical processes, including scattering, reflection, and absorption [10,11]. For these reasons, the use of PDT is limited, and it can be used for the treatment of superficial tumors only. The different drug delivery approaches should be used [12]. One of the ways to eliminate this problem is to use a method combining PDT and ionizing irradiation therapies. These electromagnetic waves can travel in the biological tissues with minimal losses [8].

The main idea of this method is that the photosensitizer molecules are bound to the scintillating nanoparticle [13]. The absorption spectrum of the photosensitizer should overlap with the emission spectrum of the nanoparticle. Further, the energy of ionizing radiation is converted into the production of singlet oxygen.

Very important requirements are imposed on the scintillating nanoparticles. Specifically, these nanoparticles should efficiently absorb ionizing radiation and convert its energy into photosensitizer excitation with the subsequent formation of singlet oxygen. 

Among a relatively wide variety of scintillating materials, rare-earth fluoride nanoparticles are considered very promising for scintillating. Indeed, the presence of rare-earth elements having high atomic numbers increases the absorption cross-section significantly [4,7,14,15]. According to the literature data [4,7], fluoride nanoparticles can be easily coated with biocompatible polymers and loaded with photosensitizers. The coated and unmodified rare-earth fluoride nanoparticles demonstrate low cytotoxicity toward many cell lines [16,17,18,19]. Materials containing cerium are considered very efficient scintillators converting ionizing irradiation into UV one [20]. In turn, the co-activation with Tb^3+^ ions allows intense green luminescence to be obtained due to an effective energy transfer from Ce^3+^ to Tb^3+^ [21,22,23]. In this system, the brightest Tb^3+^ luminescence peaks correspond to ^5^D_4_–^7^F_J_ (J = 3–6) transitions. The Tb^3+^ luminescence overlaps with the absorption of one of some clinically approved photosensitizers based on chorine [24]. For the efficient X-ray-induced PDT, the conjugation method of photosensitizers and nanoparticles is crucial. In the case of inorganic photosensitizers such as ZnO, it is possible to create double-phase nanocomposites [14,25] or core-shell structures [15]. For clinically approved organic photosensitizers, a polymeric shell is required in many cases. In particular, polyethylenimine (PIE) on the surface of Cu_2−x_S nanoparticles can be branched with chlorin (e6) [26]. Also, the possibility of Chlorin e6-based photosensitizer conjugation to polyvinylpyrrolidone (PVP) has been shown in several works [27,28,29,30]. 

However, the work demonstrates the conjugation of Tb^3+^:LaF_3_ nanoparticles with Rose Bengal via the electrostatic interaction of negatively charged functional groups of Rose Bengal photosensitizer and the positively charged Tb^3+^:LaF_3_ nanoparticle surface due to F^–^ vacancies on the surface. In turn, the Eu^3+^:BaGdF_5_/SiO_2_ core/shell nanoparticles loaded with methylene blue photosensitizer demonstrated their efficiency in X-ray-induced PDT due to the porous nature of the SiO_2_ shell [31].

The search for convenient coating polymers for conjugation with specific photosensitizers is a very challenging task in the modern scientific community.

In our previous work [24], we demonstrated the efficiency of energy transfer from CeF_3_-YF_3_-TbF_3_ nanoparticles to Radachlorin via PEI. The aim of this work was to study the spectral and kinetic characteristics of promising nanocomposites based on Ce_0.5_Y_0.35_Tb_0.15_F_3_ nanoparticles conjugated with Radachlorin using polyvinylpyrrolidone. The nonradiative energy transfer from Tb^3+^ ions to Radachlorin molecules was investigated. The toxicity of the composites to the A549 cell culture was also evaluated using the MTT test.

## 2. Materials and Methods

### 2.1. Experimental Technique

The optical excitation of the luminescence was performed via 266 nm laser radiation, using the 4th harmonic of the YAG:Nd laser from Lotis TII LS-2147 (Belarus, Minsk, Belarus), which operated in Q-switched mode (pulse duration was 10 ns). The spectra were registered using a StellarNet portable spectrometer (Tampa, FL, USA). The luminescence decay curves were registered using a monochromator with 1200 lines per mm diffraction grating. The signal was registered using a photomultiplier tube FEU100 (Russia) and digital oscilloscope Rhode&Schwartz (Munich, Germany) with a 1 GHz bandwidth. The X-ray luminescence (XRL) spectra of the prepared samples were measured on a laboratory installation built with an X-ray source (tungsten anode operating at a voltage of 40 kV and a current of 35 mA, Russia) and an FSD-10 mini-spectrometer (Optofiber LLC, Moscow, Russia). The photodynamic activity of both Radachlorin and Radachlorin—CeF_3_-YF_3_-TbF_3_ nanoparticle conjugated was studied using a commercial medical light source based on laser emitting diode Latus (Saint Petersburg, Russia) (λ_em_ = 662 nm). Physical characterization of the nanoparticles was performed with a Bruker D8 (Billerica, MA, USA) diffractometer with Cu K_α_-radiation and a Hitachi HT7700 Exalens (Tokyo, Japan) transmission electron microscope TEM) with an accelerating voltage of 100 kV in the TEM mode. The sample preparation: the suspension (10 microliters) was placed on a formvar/carbon lacey 3 mm copper grid; drying was performed at room temperature. After drying, the grid was placed in a transmission electron microscope using a special holder for microanalysis. The analysis was held at an accelerating voltage of 100 kV in TEM mode. The average diameter calculation was based on the analysis of 160 nanoparticles [32]. To get the diameter (D) of the nanoparticles, the area (in square nanometers) of each nanoparticle from the TEM image was equated to the area of a circle π⋅D^2^/4, where π = 3.14, and D is the diameter. The obtained histogram was approximated via the lognormal function where ±1 standard deviation was determined.

The colorimetric MTT assay was used to investigate the cytotoxicity of the samples with the test protocol adopted from work [33]. The A549 cells were investigated in 96-well plates (SPL Lifesciences, Pocheon, Republic of Korea) in a concentration of 10^4^ cells/well. After seeding and 12 h incubation, a medium in wells was replaced with the 100 µL of fresh, containing nanoparticles (ratio of 1/10 (*v/v*) sample suspension in water/medium for each concentration, sonicated for 10 min in a sonication bath, model ODA-LQ40, 600 W, volume 4 L). Exposure time was 24 h at 37 °C in humid air (98%) containing 5% CO_2_. Three hours before the end of the exposure period, the MTT (3-(4,5-dimethyl-2-thiazolyl)-2,5-diphenyltetrazolium bromide, Sigma-Aldrich, #M5655, St. Louis, MO, USA) solution in 0.1 M pH 7.2 phosphate-buffered saline (PBS) (5 mg/mL, 20 μL/well) was added to the cells. After the completion of the exposure period, the supernatant was removed, and 100 μL/well solution containing 10% SDS (Sigma-Aldrich, #L3771) in PBS was added. Absorbance at 570 nm for each well was measured using a microplate reader (Biorad, xMark, Moscow, Russia).

### 2.2. Synthesis of the Nanoparticles

The CeF_3_-YF_3_-TbF_3_ nanoparticles were fabricated by co-precipitation using an aqueous solution technique. It is a well-established synthesis that results in nanoparticles of good crystallinity and relatively small dimensions for CeF_3_-based compounds [34]. We used an ammonium citrate solution with a 3-fold excess of NH_4_F as a fluorinating agent. All the chemicals used were of analytical grade. Monohydrate citric acid (C_6_H_8_O_7_·H_2_O), Y(NO_3_)_3_·6H_2_O, Ce(NO_3_)_3_·6H_2_O, Tb(NO_3_)_3_·6H_2_O, NH_4_F, ammonium, and polyvinylpyrrolidone (PVP) were purchased from Sigma-Aldrich. All the chemicals were used without further purification. The concentration of the doping ions is represented in molar percentage (mol.%). The choice of the Ce_0.5_Y_0.35_Tb_0.15_F_3_ sample was based on the observation that at these ratios between the ions, the highest energy transfer rate from Ce^3+^ to Tb^3+^ occurs [35,36]. In order to form the PVP-nanoparticle composites, 100 mg of dried nanoparticles were suspended in 10 mL of distilled water via sonication. Also, 25 mL of distilled water was added to 100 mg of PVP. The mixture was placed in an ultrasonic bath (model ODALQ40, 600 W, volume 4 L) for 7 min in order to obtain a homogenous solution. The colloidal solution of the nanoparticles was added dropwise to the PVP solution and stirred for several hours. Washing by centrifugation was carried out to remove residual unreacted PVP (until the pH factor was about 6). A solution of polyvinylpyrrolidone with a mass fraction of 1% was added dropwise to the colloid of nanoparticles with continuous stirring. We used commercially available Radachlorin as a photosensitizer produced by RadaPharma company (Saint Petersburg, Russia). It consists of a 3.5 mg/mL water solution of a mixture of sodium salts of chlorine e6, chlorine p6, and purpurin 5 [37].

## 3. Results

### 3.1. Characterization of Ce_0.5_Y_0.35_Tb_0.15_F_3_ Nanoparticles

**Figure 1:**
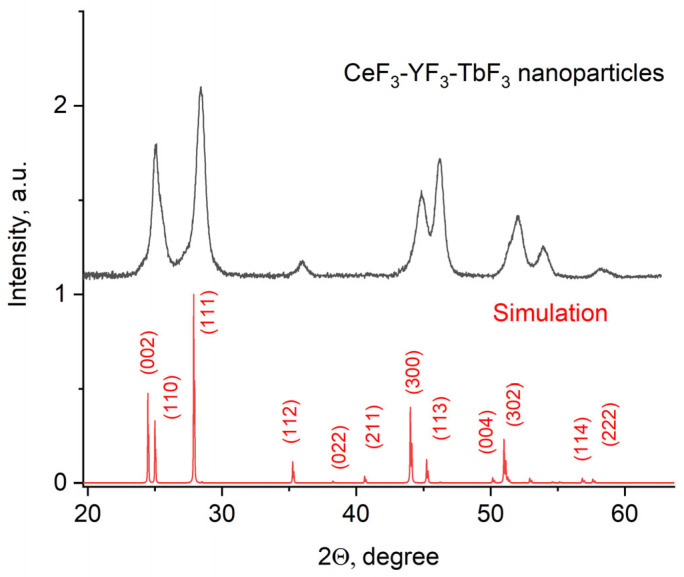
X-ray diffraction pattern of the Ce_0.5_Y_0.35_Tb_0.15_F_3_ nanoparticles.

The X-ray diffraction pattern of the Ce_0.5_Y_0.35_Tb_0.15_F_3_ powder is presented in Figure 1. 

**Figure 2:**
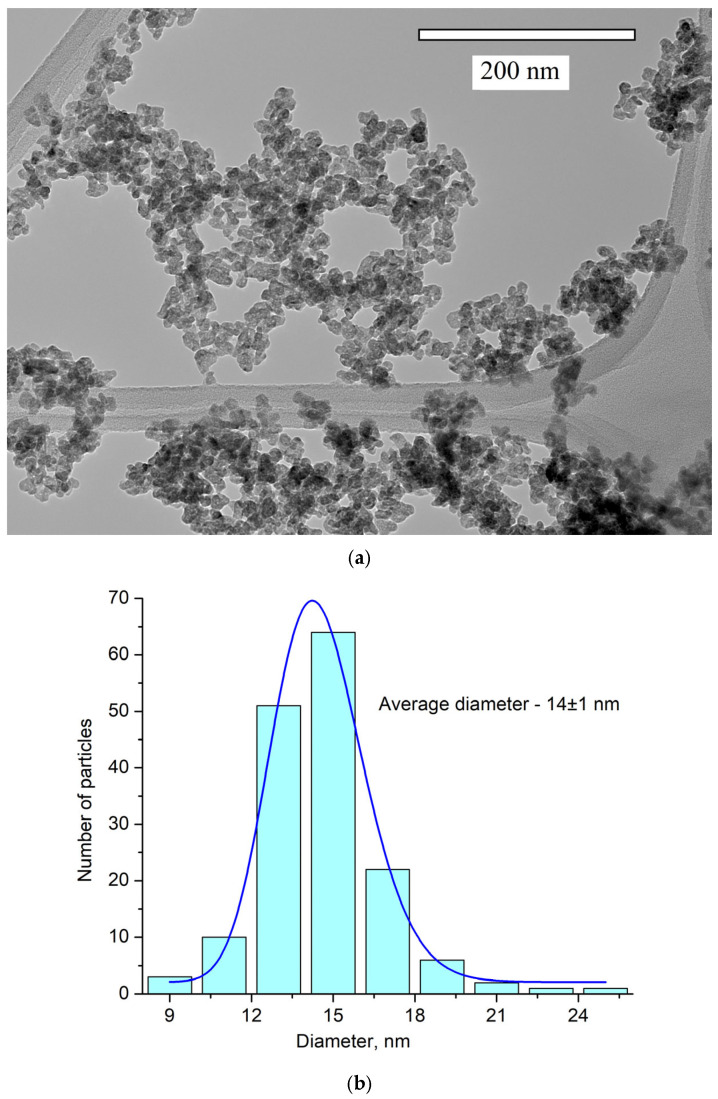
TEM image (**a**) and the size distribution histogram (**b**) of Ce_0.5_Y_0.35_Tb_0.15_F_3_ nanoparticles. The obtained histogram is approximated via the lognormal function (blue line).

The obtained pattern corresponds to the single-phase hexagonal structure of the CeF_3_ host matrix. It can be seen that the XRD pattern is shifted toward higher angles. It can be explained by the fact that Y^3+^ (0.1011 nm) has a lower ionic radius compared to Ce^3+^ (0.115). The Y^3+^ lessens the lattice parameters, and the pattern is shifted [38]. The peaks are also broadened. It can be related to the nanosized dimensionality of the samples, as well as to notable differences in the ionic radii of Ce^3+^ and Y^3+^. The crystal lattice parameters of the samples were determined, namely a = 7.02 ± 0.15 Å, c = 7.20 ± 0.14 Å [39]. The simulation was carried out using the VESTA software Version 3 [40]. TEM images of the nanoparticles and the size distribution histogram are presented in Figure 2a,b, respectively. 

The nanoparticle shape is not perfectly spherical. The average diameter of the nanoparticles is 14 ± 1 nm. Nanoparticles of this size can be used for biomedical applications [3]. 

### 3.2. Spectral-Kinetic Characteristics of Nanoparticles Ce_0.5_Y_0.35_Tb_0.15_F_3_

**Figure 3:**
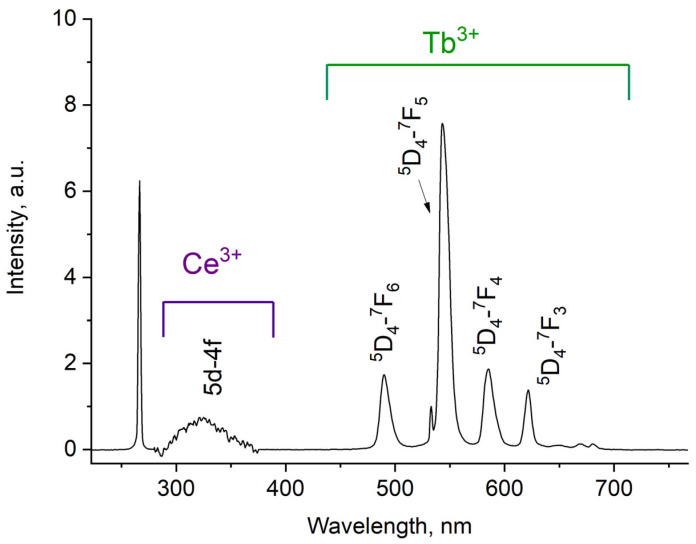
Room temperature luminescence spectra of Ce_0.5_Y_0.35_Tb_0.15_F_3_ nanoparticles as 7.9 g/L water colloidal solution. λ_ex_ = 266 nm corresponds to 4f–5d absorption band of Ce^3+^.

The luminescence spectrum of 7.9 g/L water colloidal solution (Ce_0.5_Y_0.35_Tb_0.15_F_3_ nanoparticles) under 266 nm excitation is shown in Figure 3.

**Figure 4:**
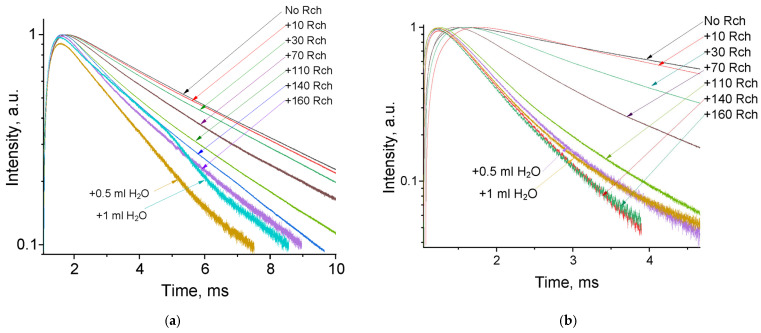

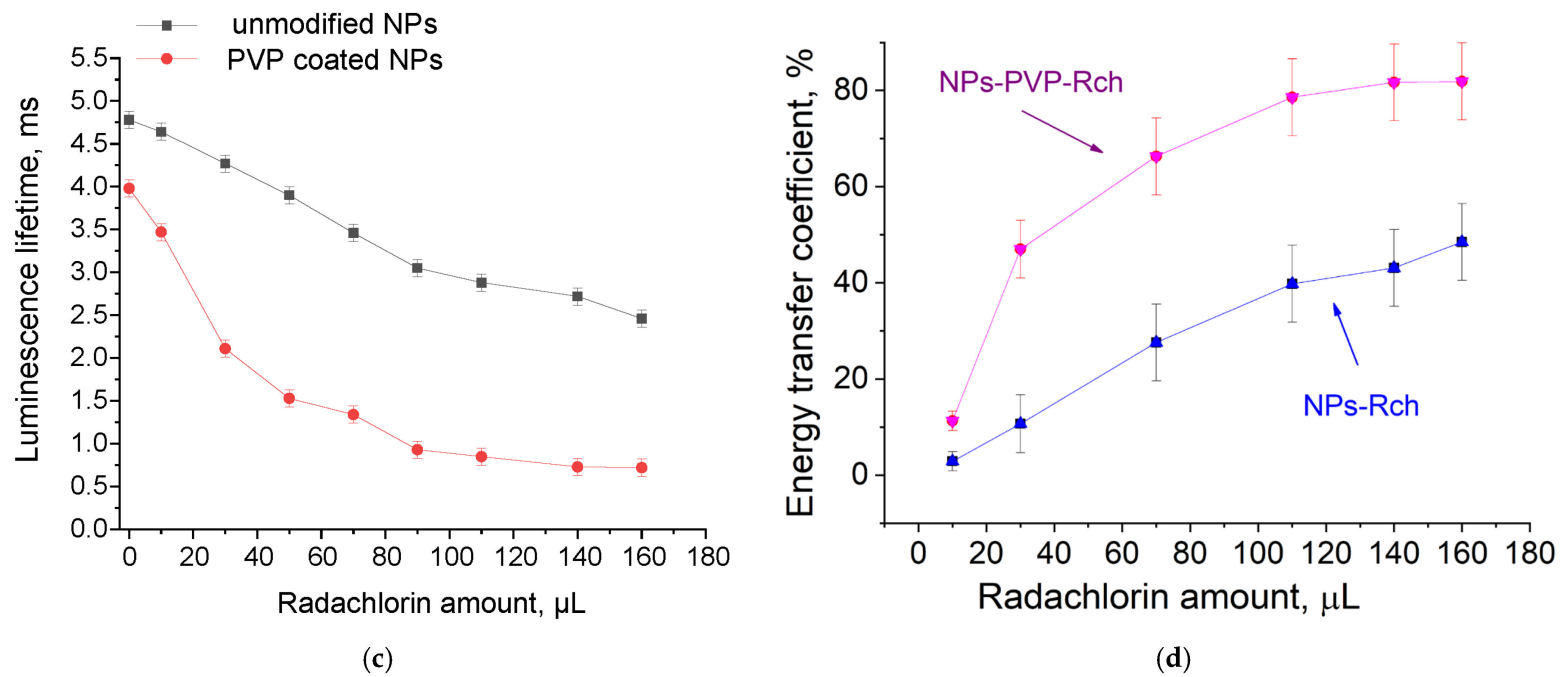
The luminescence kinetics of Tb^3+^ ions (λ_em_ = 541 nm, ^5^D_4_–^7^F_5_ transition) of PVP- coated nanoparticles (**a**) and unmodified ones (**b**), decay times at different Rch concentrations (**c**), and energy transfer coefficients (**d**).

The luminescence peaks correspond to trivalent terbium and cerium ions. Specifically, the broad luminescence band in the 280–400 nm spectral range is due to the 5d–4f transitions of Ce^3+^ ions. In turn, the peaks in the visible spectral range are 4f–4f emissions of Tb^3+^ ions (transitions from ^5^D_4_ to ^7^F_J_) [21,41]. The possibility of nanoparticle conjugation with photosensitizer molecules was studied using polyvinylpyrrolidone (PVP) as the coating material of the nanoparticles. The Radachlorin (Rch) photosensitizer was diluted in distilled water in a ratio of 6:50 (0.5 mL of water and 60 μL of Radochlorin) and added in different volumes to the colloidal solutions of nanoparticles coated with PVP and without the coating (two samples). The obtained results are presented in Figure 4. Specifically, the luminescence kinetics of Tb^3+^ ions (λ_em_ = 541 nm, ^5^D_4_–^7^F_5_ transition) of the PVP- coated nanoparticles (Figure 4a) and unmodified ones (Figure 4b), decay times at different Radachlorin concentrations (Figure 4c), and energy transfer coefficients (Figure 4d).

**Figure 5:**
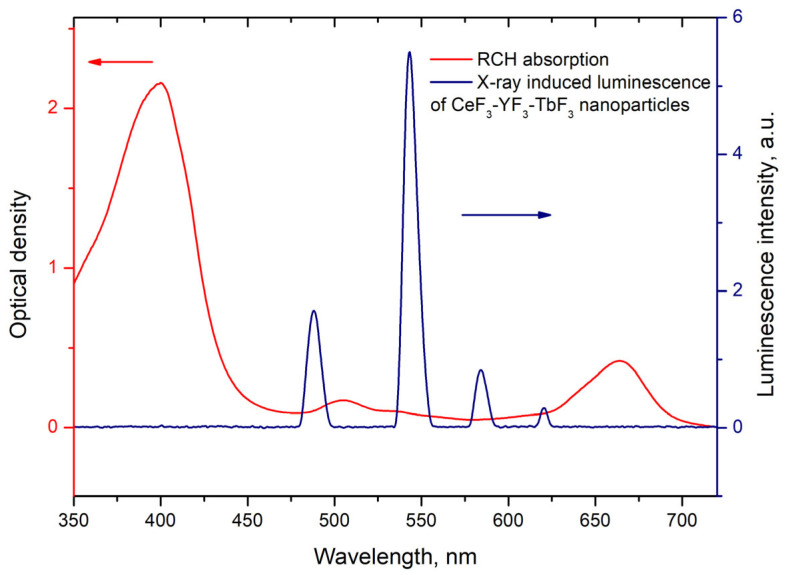
Radachlorin absorption spectrum and X-ray luminescence spectrum of Ce_0.5_Y_0.35_Tb_0.15_F_3_ nanoparticles. The arrows indicate corresponding Y-axis.

It can be seen that an increase in the concentration of Rch molecules in the colloid leads to the shortening of the luminescence decay times of Tb^3+^ ions, which is apparently a consequence of efficient nonradiative energy transfer from nanoparticles to Rch. This tendency is observed for both types of samples (Figure 4a,b). We have diluted colloids with high concentrations of Rch with distilled water to check the stability of the nanoparticle–Rch complexes. In the case of unmodified nanoparticles, the luminescence decay rate got back to the larger values. It means that this complex is not stable, and the photosensitizer molecules are capable of separating from the nanoparticle surface. In the case of nanoparticle–PVP–Rch composites, the decay curves stayed constant after the addition of water. It apparently indicates the formation of relatively stable conjugates. For higher concentrations of Rch, the deviation from the single exponential law of the decay curves is observed. This observation can be related to the fact that different nanoparticles are conjugated with different amounts of Rch molecules. In this case, the rate of Tb^3+^ luminescence quenching depends on the amount of conjugated Rch molecules; in turn, it leads to the multiexponential character of the decay curve. The luminescence lifetime was calculated as an average lifetime according to Formula (1) [42]:

(1) $$t_{av}=\frac{\int t*I(t)dt}{\int I(t)dt}$$

where $I\left(t\right)$—is the luminescence intensity. The results of the lifetime evaluation are presented in Figure 4c. The values of the luminescence lifetime of the Tb^3+^ ions at 541 nm obtained here for the colloidal solution of unmodified nanoparticles without Rch appear to be larger than those obtained by us for dry samples in our previous work [23]. It can be explained by the radiative energy transfer between nanoparticles in colloids. The efficiency of energy transfer ($k_{ET}$) can be calculated from luminescence decay time values according to Formula (2) [43]:

(2) kET=1− τNPsτNPs+Rch

where $\tau_{NPs}$ is the lifetime of the ^5^D_4_–^7^F_5_ transition of Tb^3+^ ions in the absence of Rch and $\tau_{NPs+Rch}$ is the decay time of the same conjugated with Rch. The results of the energy transfer coefficient evaluation are presented in Figure 4d. The efficiency of energy transfer for PVP-coated nanoparticles increases faster at low amounts of Rch compared to unmodified nanoparticles. The highest value of energy transfer efficiency is 80% in the solution with 140–160 µL Rch. This value lies within the trend of optimized bioconjugates and nanocomposites for combined photodynamic therapy [44]. For example, a FRET efficiency from the β-NaLuF_4_:Tb^3+^ scintillator nanoparticle to the Rose Bengal PDT agent molecule was measured as 94.29% [13]. It is possible to estimate the effective distance between Tb^3+^ ions and Rch molecules. The Forster resonance energy transfer (FRET) theory is a reliable approach for the estimation of this value [45,46]. The FRET is a strongly distance-dependent process of energy transfer between two elements (donor and acceptor). This energy transfer takes place at a distance in the 1–20 nm range [47,48]. For FRET, it is important that the donor should be an emissive molecule or particle, and the acceptor should be able to absorb the light that the donor emits. The Radachlorin absorption spectrum and X-ray luminescence spectrum of Ce_0.5_Y_0.35_Tb_0.15_F_3_ nanoparticles are presented in Figure 5.

The information concerning the spectral overlap of the Radachlorin absorption spectrum and X-ray luminescence spectrum of the Ce_0.5_Y_0.35_Tb_0.15_F_3_ nanoparticles allows obtaining the critical radius R_0_ = 4.5 nm for these conjugates [24]. Substituting the obtained efficiencies of energy transfer (k_ET_) between the studied nanoparticles and Rch dye molecules into Formula (2) [49], the distances r between them observed in the experiment were calculated. The obtained values are presented in Table 1. The absence of Ce^3+^ emission under X-ray excitation can be explained by a shorter Ce^3+^ lifetime (tens of nanoseconds) compared to the Tb^3+^ one (several milliseconds).

(3) $$k_{FRET}=\frac{1}{1+{\left(\frac{r}{R_{0}}\right)}^{6}}$$

Based on the results of the calculations, it can be concluded that with an increase in the number of Rch molecules in the colloid, the distance between nanoparticles and Rch molecules decreases. In the case of the lowest dye concentration, the distance between the nanoparticles and Radachlorin molecules is *r* = 8.1 nm, and for the nanoparticles coated with PVP, it is *r* = 6.3 nm. In the case of the highest Rch concentration, the distance between the nanoparticles and Radachlorin molecules is *r* = 4.6 nm, and for nanoparticles coated with PVP, it is *r* = 3.5 nm. Further, after adding the distilled water, the calculation of the distance between nanoparticles and Rch molecules for uncoated nanoparticles returns a slightly decreased value, which can be explained by residual agglomeration processes between molecules and nanoparticles or by an error of decay rate measurement. At the same time, with the subsequent addition of water to coated nanoparticles, this distance did not change; therefore, a stable conjugate was formed. 

### 3.3. Evaluation of the Survival of A549 Cells in the Presence of Ce_0.5_Y_0.35_Tb_0.15_F_3_ Nanoparticles and Radachlorin

**Figure 6:**
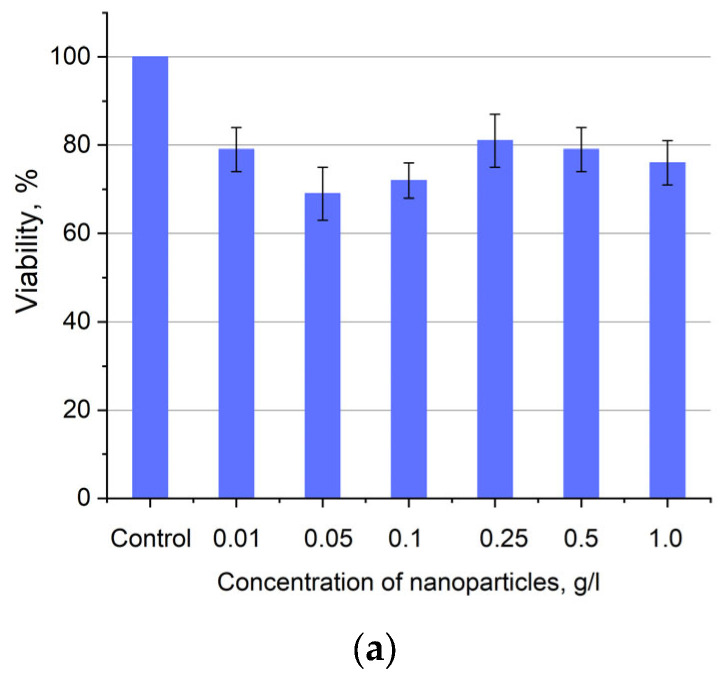

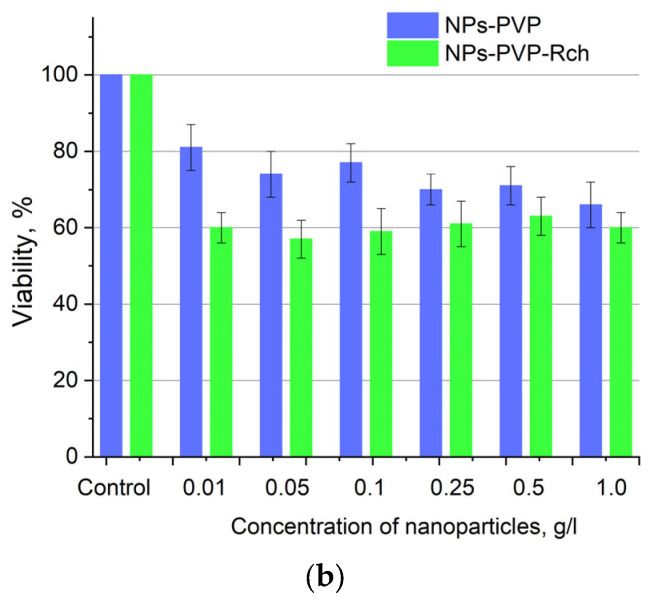
Cytotoxicity of Ce_0.5_Y_0.35_Tb_0.15_F_3_ unmodified nanoparticles (**a**) and nanoparticles coated with PVP (**b**) towards A549 cells. In diagram (**b**) (NPs–PVP—without the addition of Radachlorin; NPs–PVP Rch—with the addition of Radachlorin).

**Figure 7:**
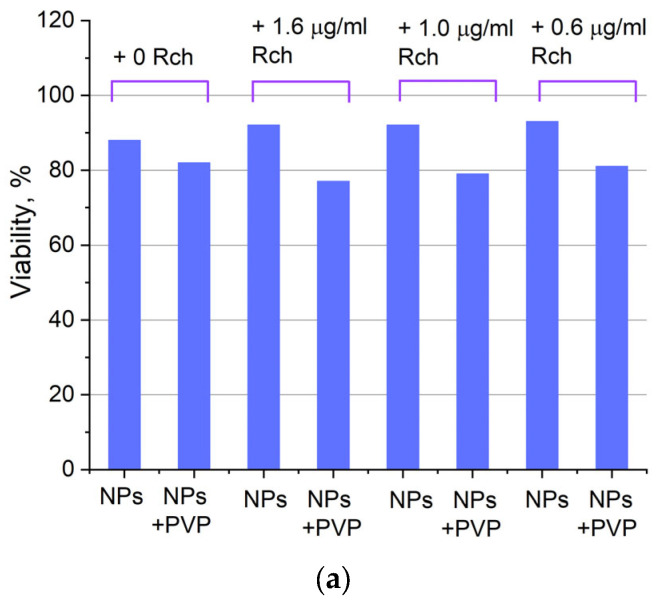

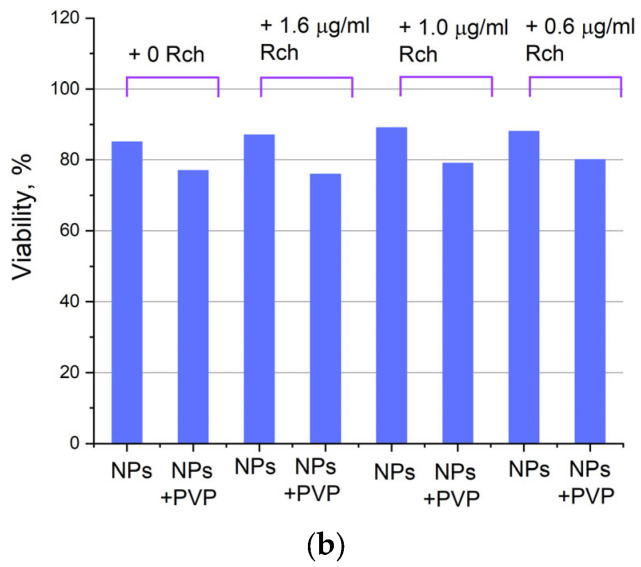
Photoinduced cytotoxicity of 0.1 mg/mL (**a**) and 0.5 mg/mL (**b**) solution of Ce_0.5_Y_0.35_Tb_0.15_F_3_ nanoparticles conjugated to Rch with PVP toward A549 cells under 266 nm laser irradiation.

Human lung carcinoma cells (A549) were used to assess the cytotoxicity of the nanoparticles. Cells were cultured in 25 µL DMEM medium supplemented with 10% inactivated fetal bovine serum, 2 mM glutamine, 100 U/mL penicillin, and 100 U/mL streptomycin in 5% CO_2_ at 37 °C in an incubator. Approximately 350,000 cells were used for the test. The expected cytotoxicity was assessed using the MTT assay. The MTT reagent was added to each well of the cell plate. The results are presented in Figure 6 and Figure 7.

**Figure 8:**
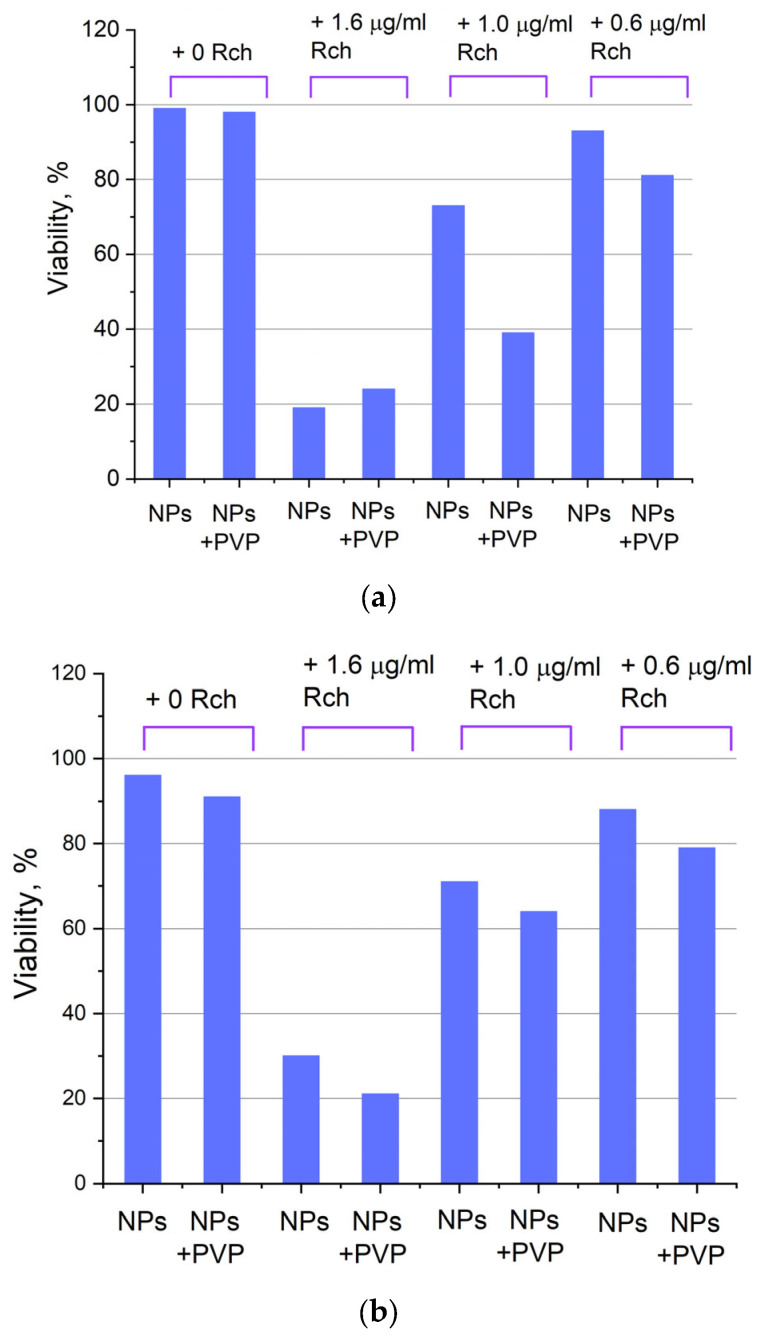
Photoinduced cytotoxicity of Ce_0.5_Y_0.35_Tb_0.15_F_3_ nanoparticles at a concentration of 0.1 mg/mL (**a**) and 0.5 mg/mL (**b**) conjugated to Rch using PVP on A549 cells under irradiation by Latus laser device.

Figure 6a shows that there is no clear survival dependence on the nanoparticle concentration. At the same time, cell survival in the case of nanoparticles coated with PVP does not fall below 50%. An area with a high concentration is characterized by an increase in survival. It can be suggested that for these concentrations, the formation of large agglomerates takes place. These large agglomerates can precipitate without interaction with cells, or the large size hinders this interaction (cell uptake). It was found that with a consistent increase in the number of Rch molecules in the colloid, the survival of A549 cells decreases (see Figure 6b). Since the experiments were not performed in complete darkness, the survival decrease can be explained by the generation of reactive oxygen species. Photoinduced cytotoxicity was also investigated. Cells with nanoparticles were irradiated with a laser at a wavelength of 266 nm (see Figure 7a,b), as well as with a Latus laser device designed for photodynamic therapy using Rch (see Figure 8).

**Figure 9:**
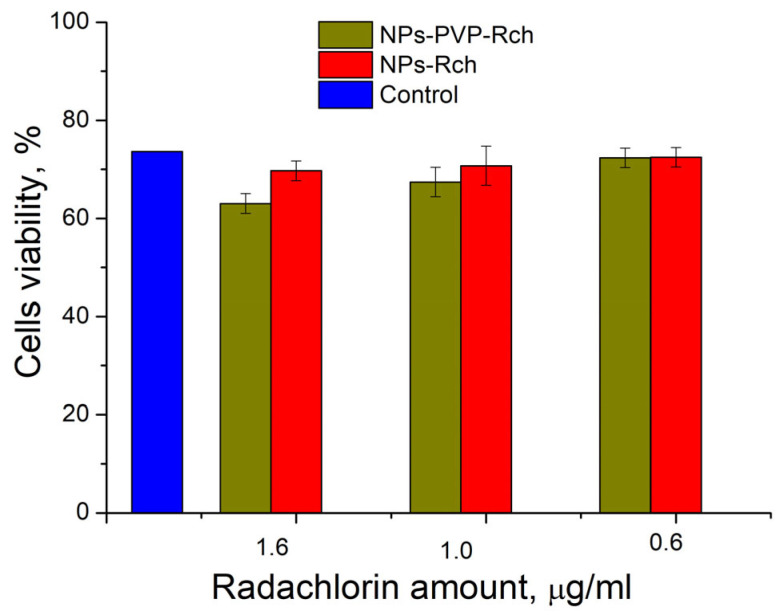
X-ray induced cytotoxicity of Ce _0.5_ Y _0.35_ Tb _0.15_ F _3_ nanoparticles at a concentration of 0.1 μg/mL conjugated to Radachlorin using polyvinylpyrrolidone on A549 cells.

It can be seen that the photodynamic activity of both coated and unmodified nanoparticles is not significant under 266 nm irradiation. It can be suggested that the DMEM biological medium absorbs and/or scatters this kind of irradiation, preventing the efficient formation of singlet oxygen. In turn, the Latus laser device provided efficient photodynamic activity of coated and unmodified samples. It can be concluded that Rch molecules conjugated to the nanoparticles do not decompose, and they are still capable of singlet oxygen generation. One of the main conclusions is that the Rch molecule binds much better with the nanoparticle in the presence of PVP. Since nonradiative energy transfer is most efficient when the dye is conjugated with a nanoparticle, this contributes to a decrease in the survival of tumor cells. We also tested the full path of energy transfer for our scintillating nanoparticles. The cytotoxicity of the samples induced by X-ray irradiation is presented in Figure 9. The toxicity was also studied towards A549 cells. The X-ray source was presented using a W-tube working at 85 kV voltages; the exposition lasted for 45 min. 

We can see that Ce_0.5_Y_0.35_Tb_0.15_F_3_-Rch composites do not work effectively. As mentioned above, unmodified nanoparticle-Rch does not demonstrate stability (Figure 4b). In turn, Ce_0.5_Y_0.35_Tb_0.15_F_3_–PVP–Rch composites provide a notable decrease in cell viability. In particular, after X-ray irradiation, the survival decreased by ~12%.

Our results show the potential of Ce_0.5_Y_0.35_Tb_0.15_F_3_–PVP–Radachlorin composites for X-ray-induced photodynamic effects on cell viability. The concentrations of NPs and photosensitizer molecules that we have used are in agreement with other works in this field, as well as efficiencies of energy transfer and effect on cell culture [13,44]. Compared with the world analogs, our system demonstrated one of the highest FRET efficiencies that can provide an efficient therapeutic effect. Indeed, the Ce_0.1_La_0.9_F_3_/LaF_3_ and CeF_3_/LaF_3_ core/shell nanoparticles conjugated with chlorin e6 showed FRET efficiency around 50% [50]. In turn, the organically modified silica nanoparticles exhibited a FRET efficiency of around 75% [51]. The Tb^3+^:LaF_3_ nanoparticles electrostatically conjugated with Rose Bengal photosensitizer demonstrated a FRET efficiency of around 85% [47]. However, it seems that Ce^3+^ ions are a more effective target for X-ray-induced PDT. There are also systems demonstrating one of the highest FRET efficiencies up to 99% (NaGdF_4_:Tb^3+^–Rose Bengal biocompatible nanocomposite [48]). At the same time, we see very moderate toxicity of investigated composites towards the cells themselves. We believe that the X-ray-induced effect on viability can be improved by optimizing the conditions of X-ray irradiation and the parameters of the X-ray source. And energy transfer from the scintillator nanoparticle to the photosensitizer can be improved by the proper selection of photosensitizer content. As can be seen from Figure 8b, the directly induced action did not lead to 100% efficiency.

## 4. Conclusions

The studied Ce_0.5_Y_0.35_Tb_0.15_F_3_ nanoparticles were synthesized by co-precipitation using an aqueous solution technique. All of the samples had the expected CeF_3_ hexagonal structure with an average nanoparticle diameter of around 14 ± 1 nm. The luminescence decay curves of Ce_0.5_Y_0.35_Tb_0.15_F_3_ nanoparticles (λ_em_ = 541 nm, ^5^D_4_–^7^F_5_ transition of Tb^3+^) conjugated with Radachlorin using polyvinylpyrrolidone coating as well as without Radachlorin were detected. Efficient nonradiative energy transfer from Tb^3+^ to the Radachlorin was demonstrated. The maximum energy transfer coefficient for nanoparticles conjugated with Radachlorin via polyvinylpyrrolidone was approximately 82%, and without the coating, it was about 55%. Presumably, the dye molecule with the nanoparticle binds much better in the presence of polyvinylpyrrolidone. The average distance between the nanoparticle surface and Radachlorin was *R*_0_ = 4.5 nm. An increase in Radachlorin concentration led to the shortening of the distance between the nanoparticles and Radachlorin. After the addition of water, this distance does not change (only for PVP-coated samples). It means that the conjugates are stable in our experimental conditions. The cytotoxicity of the composites was assessed using the MTT test, which revealed that with a consistent increase in the number of Radachlorin molecules in the colloid, the survival of A549 cells declines. The best results for X-ray-induced cytotoxicity were observed for the NP–PVP–Rch sample at the lowest Rch concentration. In particular, after X-ray irradiation, the survival rate decreased by 12%. 

## Figures and Tables

**Table 1:** Distances between Ce_0.5_Y_0.35_Tb_0.15_F_3_ nanoparticles and Radachlorin molecules.

Amount of Rch, µL	Distance r, nm	
	Unmodified	Coated with PVP
10	8.1 ± 1.2	6.3 ± 1.2
30	6.4 ± 1.2	4.6 ± 1.2
50	5.8 ± 1.2	4.2 ± 1.2
70	5.9 ± 1.2	5.0 ± 1.2
90	5.0 ± 1.2	3.7 ± 1.2
110	4.8 ± 1.2	3.6 ± 1.2
140	4.7 ± 1.2	3.5 ± 1.2
160	4.6 ± 1.2	3.5 ± 1.2
160 µL Rch + 0.5 mL H_2_O	4.3 ± 1.2	3.5 ± 1.2
160 µL Rch + 1.0 mL H_2_O	5.0 ± 1.2	3.5 ± 1.2

## Data Availability

Data are contained within the article.
